# Supplementary material for: Molecular Mechanisms of the Interactions of N-(2-Hydroxypropyl)methacrylamide Copolymers Designed for Cancer Therapy with Blood Plasma Proteins
Source: Pharmaceutics. 2020 Jan 28;12(2):106. doi: 10.3390/pharmaceutics12020106 (PMC7076460; doi:10.3390/pharmaceutics12020106)
Supplement: Supplementary file 1 [file pharmaceutics-12-00106-s001.pdf]

pharmaceutics

Supplementary Materials: Molecular Mechanisms of the Interactions of *N*-(2-hydroxypropyl)methacrylamide Copolymers Designed for Cancer Therapy with the Blood Plasma Proteins

Larisa Janisova, Andrey Gruzinov, Olga V. Zaborova, Nadiia Velychkivska, Ondřej Vaněk, Petr Chytil, Tomáš Etrych, Olga Janoušková, Xiaohan Zhang, Clement Blanchet, Christine M. Papadakis, Dmitri I. Svergun and Sergey K. Filippov

1. Materials. 1-Aminopropan-2-ol, methacryloyl chloride, 6-aminohexanoic acid, *N*,*N*′-dicyclohexylcarbodiimide, *tert*-butylcarbazate, *tert*-butylalcohol, trifluoroacetic acid, 4-(dimethylamino)pyridine, cholesterol, dimethyl sulfoxide, dimethylacetamide, *N*,*N*-diisopropylethylamine and 2-cyanopropan-2-yl benzodithioate, human serum albumin (HSA), fibrinogen (Fbg) and apolipoprotein (Apo) E4 and A1, immunoglobulin G (IgG), sodium dodecyl sulfate and all other chemicals were purchased from Sigma-Aldrich, Czech Republic. 2,2'-Azobis(4-methoxy-2,4-dimethylvaleronitrile) (V-70) was purchased from Fujifilm Wako Chemicals Europe, Germany. Human plasma was obtained from the Military University Hospital in Prague from healthy donors. All other chemicals and solvents were of analytical grade. The solvents were dried and purified by conventional procedures.

2. Monomers. *N*-(2-hydroxypropyl)methacrylamide (HPMA), 1-(*tert*-butoxycarbonyl)-2-(6-methacrylamido hexanoyl)hydrazine (MA-Ahx-NHNH-Boc) and cholest-5en-3β-yl 6-methacrylamido hexanoate (MA-Ahx-Chol) were synthesized as described previously. [15–17]

3. Synthesis and characterization of the copolymer. The copolymer pHPMA-Chol was prepared by RAFT polymerization of HPMA, MA-Ahx-NHNH-Boc and MA-Ahx-Chol using V-70 as an initiator, 2-cyanopropan-2-yl benzodithioate as the RAFT chain transfer agent (CTA), and the mixture of dimethylacetamide:*tert*-butylalcohol (1:9) as the solvent. The reaction condition was adapted from reference [16]. The molar ratio of monomers:CTA:V-70 was 350:2:1. The molar ratio of HPMA : MA-Ahx-NHNH-Boc : MA-Ahx-Chol was 89.5 : 8 : 2.5. The total concentration of monomers was 0.7 M. The reaction mixture was bubbled with argon, sealed in ampules and the polymerization was carried out during 16 h at 40 °C. The polymer was isolated by precipitation into the mixture of acetone:diethylether 3:1, dissolved in methanol and precipitated into acetone:diethylether 3:1, filtered and dried to constant weight. Then the polymers were dissolved in DMSO and kept at 80 °C for 2 h to remove the dithiobenzoate end groups. The polymers were isolated by the same procedure as described above. At final step, the hydrazide groups were deprotected by trifluoracetic acid as described before [16]. The yield was 75%.

Molecular weight was determined by HPLC Shimadzu system with a GPC column (TSKgel Super SW3000, 300 × 4.6 mm; 4 μm) and the photo-diode array, differential refractive index (Optilab-rEX) and multiangle light scattering (DAWN HELEOS II) (both Wyatt Technology Co.) detectors. The eluent was methanol/0.3 M sodium acetate buffer, pH 6.5 (80:20 *v*/*v*) at a flow-rate of 0.3 mL min^{-1}. The values were calculated using ASTRA VI software and refractive increment index *dn*/*dc* 0.175 mL g^{-1} was used. The Optilab-rEX detector enables direct determination of sample *dn*/*dc* and solvent refractive index provides 100 % recovery of the injected sample from the column. Amount of monomer units bearing cholesterol in the copolymer was determined using ^{1}H-NMR spectroscopy in DMSO as the ratio between the characteristic shifts for HPMA and cholesterol as described in reference.[15] The hydrodynamic radius of NP in phosphate-buffered saline (0.01 g · mL^{-1}; pH 7.4) was measured using a Nano-ZS instrument (ZEN3600, Malvern, UK). The intensity of the scattered light was detected at angle θ = 173° using a laser with a wavelength of 632.8 nm. To evaluate the dynamic light scattering data, the DTS(Nano) program was used. The value was a mean of at least five independent measurements and was not extrapolated to zero concentration.

3. SAXS measurements. Solutions of proteins, pHPMA and mixtures protein/pHPMA were measured at 37 °C. Most of the samples had no measurable radiation damage detected by comparison of these frames. The measured two-dimensional scattering patterns were azimuthally averaged to yield the scattered intensity I(s) as a function of the momentum transfer s (Figure S1). Primary data processing was performed with SASFLOW pipeline. [30,31]

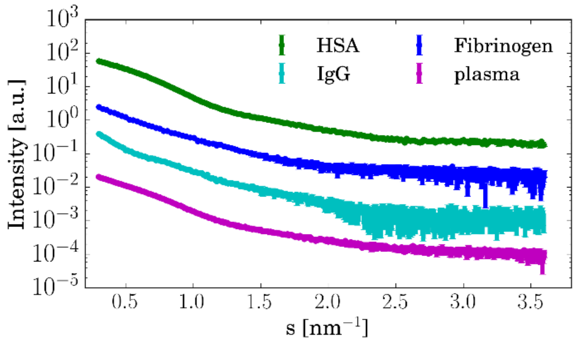

Figure S1. The dependence of the scattered intensity I(s) on the momentum transfer s for HSA, fibrinogen, IgG and plasma. Curves are shifted for better representation.

All data manipulations were performed using the PRIMUS the low-resolution particle shapes of the NPs were reconstructed by DAMMIF. The results of 10 DAMMIF runs were averaged to determine common structural features using DAMAVER and to obtain the search volume for the final ab initio shape reconstruction with DAMMIN. [30,31] To assess the possible interactions between NPs and proteins scattering curves from individual components were compared to those from their mixtures. Generally, in the absence of interactions between the components the scattering from a mixture is expressed as a sum of the contributions from the components. [27] The SAXS curves from the mixed solutions could then be fitted by a linear combination of two terms, i.e. the scattering from pHPMA-Chol and from individual plasma proteins or plasma itself (PP):

$$I(s) = \nu_{pHPMA} I_{pHPMA}(s) + \nu_{PP} I_{PP}(s), \tag{S1}$$

where ν_{pHPMA}, ν_{PP}, $I_{pHPMA}(s)$ and $I_{PP}(s)$ denote the volume fractions and the scattering intensities of the components.

To solve Eq (S1) with respect the volume fractions, program OLIGOMER was used minimizing the discrepancy between the experimental scattering I(s) from the mixture and the calculated curve:

$$\chi^2 = \frac{1}{N-1}\sum_j \left[\frac{I(s_j) - cI_{calc}(s_j)}{\sigma(s_j)}\right]^2, \tag{S2}$$

where N is the number of experimental points, c is a scaling factor, $I_{calc}(s_j)$ and $\sigma(s_j)$ are the calculated intensity and the experimental error at the momentum transfer s_j, respectively [22].

If the linear combination Eq (S1) of the scattering by components fits the experimental data from the mixture without systematic deviations, this points to the absence of interactions between the components (see example in Figure S2). If complexes are formed, the representation in Eq (S1) will not fit the experimental data and systematic deviations are observed. This approach was used to assess the presence of interactions between NPs and different plasma proteins in the mixtures studied.

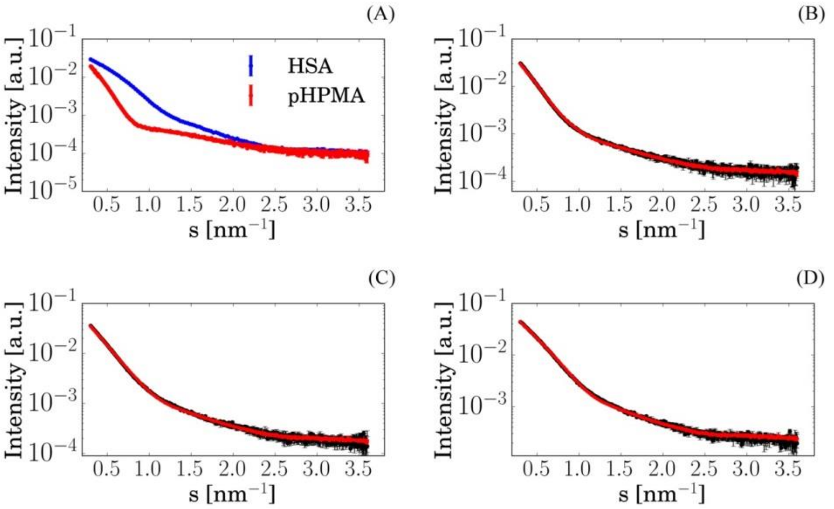

Figure S2. Analysis of pHPMA NPs mixed with HSA at different NPs/HSA ratios. Each curve was analyzed as a linear combination of two pure components measured separately. A) SAXS curves of HSA and NPs. B) pHPMA/HSA=18/5 C) pHPMA/HSA=18/10 D) pHPMA/HSA=18/20.

5. Analytical ultracentrifugation. Sedimentation velocity experiments for the sample with lower HSA concentration and for samples of free NPs were carried out at 50,000 rpm and 280 nm, while the sample with a high HSA concentration and its mixtures with NPs were analyzed at 42,000 rpm and 330 nm. In both cases, 200 absorbance scans with 30 μm spatial resolution were recorded in 4 min intervals at 20 °C; sedimentation data were also acquired using interference optics simultaneously. The samples of apolipoprotein A1 (0.3 mg/ml), apolipoprotein E4 (0.76 mg/ml), free pHPMA-Chol NPs (1 mg/ml), and their mixtures (prepared to have the same concentrations of both components as above) were analyzed at 48,000 rpm and 20°C; in total 100 scans were recorded in 6 min intervals using 230 nm absorbance signal. This wavelength corresponds to total absorbance of proteins and NPs in case of their mixtures. The samples of fibrinogen (1 mg/ml), IgG (1 mg/ml), and their mixtures with pHPMA-Chol NPs (1 mg/ml) were analyzed at 48,000 rpm and 20°C; in total 200 scans were recorded in 5 min intervals using 290 nm absorbance signal where only the proteins absorb. The buffer density and protein partial specific volumes were estimated in SEDNTERP. [30] Data were analyzed with SEDFIT software using a c(s) continuous size distribution model. [33]

Individual HSA protein and pHPMA-Chol NPs as well as their mixtures were analyzed at given concentrations (Figure S3). The absence of higher sedimentation coefficient species in the protein-NPs mixtures when compared to distributions of individual mixture components proves the lack of corona formation.

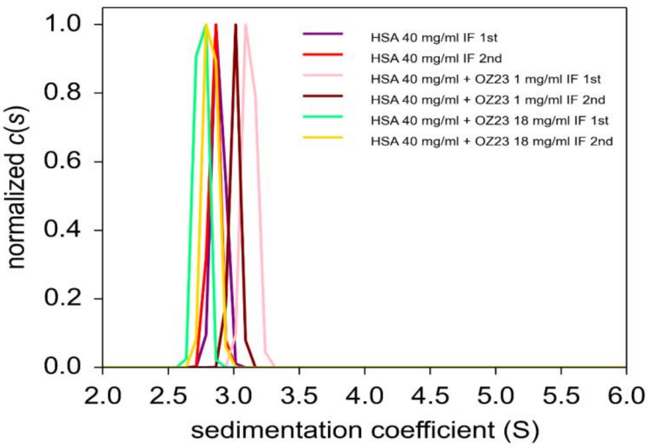

Figure S3. Normalized sedimentation coefficient distribution of the sedimenting species.

References

1. Spicer, C.D.; Jumeaux, C.; Gupta, B.; Stevens, M.M. Peptide and protein nanoparticle conjugates: Versatile platforms for biomedical applications. *Chem. Soc. Rev.* **2018**, *47*, 3574–3620.
2. Liu, Z.H.; Jiao, Y.P.; Wang, T.; Zhang, Y.M.; Xue, W. Interactions between solubilized polymer molecules and blood components. *J. Controlled Release* **2012**, *160*, 14–24.
3. Aggarwal, P.; Hall, J.B.; McLeland, C.B.; Dobrovolskaia, M.A.; McNeil, S.E. Nanoparticle interaction with plasma proteins as it relates to particle biodistribution, biocompatibility and therapeutic efficacy. *Adv. Drug Delivery Rev.* **2009**, *61*, 428–437.
4. Kim, J.A.; Aberg, C.; Salvati, A.; Dawson, K.A. Role of cell cycle on the cellular uptake and dilution of nanoparticles in a cell population. *Nat. Nanothech.* **2012**, *7*, 62–68.
5. Cai, R.; Chen, C. The Crown and the Scepter: Roles of the Protein Corona in Nanomedicine. *Adv. Mater*. **2019**, *31*, 1805740–1805752.
6. Bertrand, N.; Grenier, P.; Mahmoudi, M.; Lima, E.M.; Appel, E.A.; Dormont, F.; Lim, J.M.; Karnik, R.; Langer, R.; Farokhzad, O.C. Mechanistic understanding of in vivo protein corona formation on polymeric nanoparticles and impact on pharmacokinetics. *Nat. Commun*. **2017**, *8*, 777.
7. Zhao, Z.; Ukidve, A.; Krishnan, V.; Mitragotri, S. Effect of Physicochemical and Surface Properties on In Vivo Fate of Drug Nanocarriers. *Adv. Drug Delivery Rev*. **2019**, *143*, 3–21.
8. Thiele, L.; Diederichs, J.E.; Reszka, R.; Merkle, H.P.; Walter, E. Competitive adsorption of serum proteins at microparticles affects phagocytosis by dendritic cells. *Biomaterials* **2003**, *24*, 1409–1418.
9. Xiao, W.; Xiong, J.; Zhang, S.; Xiong, Y.; Zhang, H.; Gao, H. Influence of ligands property and particle size of gold nanoparticles on the protein adsorption and corresponding targeting ability. *Int. J. Pharm.* **2018**, *538*, 105–111.
10. Barriga, H.M.G.; Holme, M.N.; Stevens, M.M. Cubosomes: The Next Generation of Smart Lipid Nanoparticles? *Angew. Chem. Int. Ed*. **2019**, *58*, 2958–2978.
11. Lu, X.; Xu, P.P.; Ding, H.M.; Yu, Y.S.; Huo, D.; Ma, Y.Q. Tailoring the component of protein corona via simple chemistry. *Nat. Commun.* **2019**, *10*, 4520.
12. Rabanel, J.M.; Adibnia, V.; Tehrani, S.F.; Sanche, S.; Hildgen, P.; Banquy, X.; Ramassamy, C. Nanoparticle Heterogeneity: An Emerging Structural Parameter Influencing Particle Fate in Biological Media? *Nanoscale* **2019**, *11*, 383–406.
13. Zhdanov, V.P. Nanoparticles without and with protein corona: Van der Waals and hydration interaction. *J. Biol. Phys.* **2019**, *45*, 307–316.
14. Fisher, K.D.; Seymour, L.W. HPMA copolymers for masking and retargeting of therapeutic viruses. *Adv. Drug Delivery Rev.* **2010**, *62*, 240–245.
15. Chytil, P.; Etrych, T.; Koňák, Č.; Šírová, M.; Mrkvan, T.; Bouček, J.; Říhová, B.; Ulbrich, K. New HPMA copolymer-based drug carriers with covalently bound hydrophobic substituents for solid tumour targeting. *J. Controlled Release* **2008**, *127/2*, 121–130,
16. Chytil, P.; Etrych, T.; Jaroslav, K.; Šubr, V.; Ulbrich, K. *N*-(2-Hydroxypropyl)Methacrylamide-Based Polymer Conjugates with pH-Controlled Activation of Doxorubicin for Cell-Specific or Passive Tumour Targeting. Synthesis By Raft Polymerisation and Physicochemical Characterisation. *Eur. J. Pharm. Sci.* **2010**, *41*, 473–482.
17. Ulbrich, K.; Etrych, T.; Chytil, P.; Jelínková, M.; Říhová, B. Antibody-targeted polymer-doxorubicin conjugates with pH-controlled activation. *J. Drug Target.* **2004**, *12*, 477–489.
18. Zhang X., Chytil P., Etrych T., Kiu W., Rodrigues L., Winter G., Filippov S. K., Papadakis C. M. Binding of HSA to Macromolecular pHPMA Based Nanoparticles for Drug Delivery: An Investigation Using Fluorescence Methods. *Langmuir* **2018**, *34*, 7998–8006.
19. Filippov, S.K.; Franklin, J.M.; Konarev, P.V.; Chytil, P.; Etrych, T.; Bogomolova, A.; Dyakonova, M.; Papadakis, C.; Radulescu, A.; Ulbrich, K.; et al. Hydrolytically Degradable Polymer Micelles for Drug Delivery: A SAXS/SANS Kinetic Study. *Biomacromolecules* **2013**, *14*, 4061–4070.
20. Filippov, S.K.; Chytil, P.; Konarev, P.V.; Dyakonova, M.; Papadakis, C.; Zhigunov, A.; Plestil, J.; Stepanek, P.; Etrych, T.; Ulbrich, K.; et al. Macromolecular HPMA-Based Nanoparticles with Cholesterol for Solid-Tumor Targeting: Detailed Study of the Inner Structure of a Highly Efficient Drug Delivery System. *Biomacromolecules* **2012**, *13*, 2594–2604.
21. Zaborova, O.V.; Filippov, S.K.; Chytil, P.; Kovačik, L.; Yaroslavov, A.A.; Etrych, T. A novel approach to increase the stability of liposomal containers via in prep coating by

poly[*N*-(2-hydroxypropyl)methacrylamide] with covalently attached cholesterol groups. *Macromol. Chem. Phys.* **2018**, *219*, 1700508.
22. Zhang, X.; Niebuur, B.-J.; Chytil, P.; Etrych, T.; Filippov, S.K.; Kikhney, A.; Wieland, F.; Svergun, D.I.; Papadakis, C.M. Macromolecular pHPMA-Based Nanoparticles with Cholesterol for Solid Tumor Targeting: Behavior in HSA Protein Environment. *Biomacromolecules* **2018**, *19*, 470–480.
23. Klepac, D.; Kostková, H.; Petrova, S.; Chytil, P.; Etrych, T.; Kereïche, S.; Raška, I.; Weitz, D.A.; Filippov, S.K. Interaction of spin-labeled HPMA-based nanoparticles with human blood plasma proteins-the introduction of protein-corona-free polymer nanomedicine. *Nanoscale* **2018**, *10*, 6194–6204.
24. Calzolai, L.; Franchini, F.; Gilliland, D.; Rossi, F. Protein–Nanoparticle Interaction: Identification of the Ubiquitin-Gold Nanoparticle Interaction Site. *Nano Lett.* **2010**, *10*, 3101–3105.
25. Lundqvist, M.; Sethson, I.; Jonsson, B.H. Protein adsorption onto silica nanoparticles: Conformational changes depend on the particles' curvature and the protein stability. *Langmuir* **2004**, *20*, 10639–10647.
26. Franke, D.; Kikhney, A.G.; Svergun, D.I. Automated acquisition and analysis of small angle X-ray scattering data. *Nucl. Instrum. Methods Phys. Res. Sect. A* **2012**, *689*, 52–59.
27. Volkov, V.; Svergun, D.I. Uniqueness of ab initio shape determination in small-angle scattering. *J. Appl. Crystallogr.* **2003**, *36*, 860–864.
28. Franke, D.; Petoukhov, M.V.; Konarev, P.V.; Panjkovich, A.; Tuukkanen, A.; Mertens, H.D.T.; Kikhney, A.G.; Hajizadeh, N.R.; Franklin, J.M.; Jeffries, C.M.; et al. ATSAS 2.8: A comprehensive data analysis suite for small-angle scattering from macromolecular solutions. *J. Appl. Crystallogr.* **2017**, *50*, 1212–1225.
29. Planken, K.L.; Cölfen, H. Analytical ultracentrifugation of colloids. *Nanoscale* **2010**, *2*, 1849–1869.
30. Brown, P.H.; Schuck, P. Macromolecular size-and-shape distributions by sedimentation velocity analytical ultracentrifugation. *Biophys. J.* **2006**, *90*, 4651–4661.
31. Konarev, P.V.; Volkov, V.V.; Sokolova, A.V.; Koch, M.H.J.; Svergun, D.I. PRIMUS: A Windows PC-based system for small-angle scattering data analysis. *J. Appl. Cryst.* **2003**, *36*, 1277–1282.
32. Franke, D.; Svergun, D.I. DAMMIF, a program for rapid ab-initio shape determination in small-angle scattering. *J. Appl. Crystallogr.* **2009**, *42*, 342–346.
33. Schuck, P. Size-distribution analysis of macromolecules by sedimentation velocity ultracentrifugation and Lamm equation modeling. *Biophys. J.* **2000**, *78*, 1606–1619.
34. Mayer, M.; Meyer, B. Group epitope mapping by saturation transfer difference NMR to identify segments of a ligand in direct contact with a protein receptor *J. Am. Chem. Soc.* **2001**, *123*, 6108–6117.
35. Reddy, R.R.; Shanmugam, G.; Madhan, B. Kumar BVNP Selective binding and dynamics of imidazole alkyl sulfate ionic liquids with human serum albumin and collagen—A detailed NMR investigation. *Phys. Chem. Chem. Phys.* **2018**, *20*, 9256–9268.
36. Salvi, N.; Abyzov, A.; Blackledge, M. Analytical Description of NMR Relaxation Highlights Correlated Dynamics in Intrinsically Disordered Proteins. *Angew. Chem. Int. Ed.* **2017**, *56*, 14020–14024.
37. Hofmann, C.; Schönhoff, M. Do additives shift the LCST of poly(*N*-isopropylacrylamide) by solvent quality changes or by direct interactions? *Colloid Polym. Sci.* **2009**, *290/8*, 689–698.
